# P29 - Intestinal helminth Enterobius vermicularis as an immunomodulator factor

**DOI:** 10.1186/2045-7022-4-S1-P84

**Published:** 2014-02-28

**Authors:** Giannoula Patsantara, Evangelia-Thephano Piperaki, Chryssa Tzoumaka-Bakoula, Maria Kanariou

**Affiliations:** 1 1st Department of Pediatrics, University of Athens Medical School, “Aghia Sophia” Children’s Hospital, Athens, Greece; 2 Department of Microbiology, University of Athens Medical School, Athens, Greece; 3 2nd Department of Pediatrics, University of Athens Medical School, “P. and A. Kyriakou” Children’s Hospital, Athens, Greece; 4 Department of Immunology-Histocompatibility, “Aghia Sophia” Children's Hospital, Athens, Greece

## Background

A growing body of evidence shows an inverse association between helminthic infestation and expression of allergy. It has been suggested that *Enterobius vermicularis*, the least pathogenic human intestinal nematode and the last one in westernized societies may have a modulatory effect on the human immune system.

## Methods

Healthy Greek schoolchildren were investigated for *E. vermicularis* eggs with the adhesive-tape test*.* Sixty four of those, who were found parasitized and 67 matched controls were further assessed, using hematological and serological immune parameters, such as Eosinophil number (Eo count), serum Eosinophilic Cationic Protein (ECP), total and specific Immunoglobulin E (ΙgΕ) and the ratio of ECP/Eo count. In addition, certain cytokines were measured in parasitized children. These parameters were compared between not-parasitized and parasitized children, taking into account their atopic status, as well as history of clinically expressed allergic diseases.

## Results

Eo count, ECP and IgE levels were found higher in parasitized than in not-parasitized children (p≤.035 for all) indicating a type 2 immune response activation during infestation. Eo count and IgE were found significantly higher in the atopic group, whereas Eo count and ECP in the nonatopic one. However, the ECP/Eo count ratio did not significantly differ between the groups compared.As expected, atopic parasitized children exhibited higher serum IgE levels (p=.001) compared to nonatopic ones, although their IL-4 levels were paradoxically lower (p=.030). ECP was found lower (p=.016) in atopic children with a history of allergic disease than in those without such history, possibly indicating immunosuppression in the former group.

## Conclusions

The results provide evidence that *E. vermicularis* elicits a protective Th2 oriented response, irrespective of the children’s atopic status. The parasite seems to contribute to an environment which might downregulate immune responses in atopic subjects, more so in those with a history of allergic disease.

